# Efficient production of soluble recombinant single chain Fv fragments by a *Pseudomonas putida *strain KT2440 cell factory

**DOI:** 10.1186/1475-2859-10-11

**Published:** 2011-02-21

**Authors:** Thorben Dammeyer, Miriam Steinwand, Sarah-C Krüger, Stefan Dübel, Michael Hust, Kenneth N Timmis

**Affiliations:** 1 Environmental Microbiology Laboratory, Helmholtz Centre for Infection Research, Inhoffenstr. 7, 38124 Braunschweig, Germany; 2 Institut für Biochemie und Biotechnologie, Technische Universität Braunschweig, Spielmannstr. 7, 38106 Braunschweig, Germany; 3 Institut für Mikrobiologie, Technische Universität Braunschweig, Spielmannstr. 7, 38106 Braunschweig, Germany

## Abstract

**Background:**

Recombinant antibody fragments have a wide range of applications in research, diagnostics and therapy. For many of these, small fragments like single chain fragment variables (scFv) function well and can be produced inexpensively in bacterial expression systems. Although *Escherichia coli *K-12 production systems are convenient, yields of different fragments, even those produced from codon-optimized expression systems, vary significantly. Where yields are inadequate, alternative production systems are needed. *Pseudomonas putida *strain KT2440 is a versatile biosafety strain known for good expression of heterologous genes, so we have explored its utility as a cell factory for production of scFvs.

**Results:**

We have generated new broad host range scFv expression constructs and assessed their production in the *Pseudomonas putida *KT2440 host. Two scFvs bind either to human C-reactive protein or to mucin1, proteins of significant medical diagnostic and therapeutic interest, whereas a third is a model anti-lysozyme scFv. The KT2440 antibody expression systems produce scFvs targeted to the periplasmic space that were processed precisely and were easily recovered and purified by single-step or tandem affinity chromatography. The influence of promoter system, codon optimization for *P. putida*, and medium on scFv yield was examined. Yields of up to 3.5 mg/l of pure, soluble, active scFv fragments were obtained from shake flask cultures of constructs based on the original codon usage and expressed from the *Ptac *expression system, yields that were 2.5-4 times higher than those from equivalent cultures of an *E. coli *K-12 expression host.

**Conclusions:**

*Pseudomonas putida *KT2440 is a good cell factory for the production of scFvs, and the broad host range constructs we have produced allow yield assessment in a number of different expression hosts when yields in one initially selected are insufficient. High cell density cultivation and further optimization and refinement of the KT2440 cell factory will achieve additional increases in the yields of scFvs.

## Background

Recombinant antibodies and antibody fragments are indispensable tools for research, diagnostics and therapy [1-5]. Complete and natively glycolsylated antibodies, like IgGs, needed for therapeutic purposes must thus far be produced in mammalian cells. However, although yields from mammalian cells tend to be good, production times and costs are high [6]. However, for many non-therapeutic applications, and also some therapeutic applications for which effector functions are not necessary, antibody modules, such as single chain fragment variable (scFv) and fragment antigen binding (Fab) are sufficient [7,8]. Because of their lower costs and faster production cycles, microbial systems are more attractive than mammalian cell systems for the production of antibody fragments. The folding and export of scFvs in Gram-negative systems is usually more efficient than that of Fab fragments [9]. One problem with prokaryotic production systems is that expression levels of fragments of different antibodies tend to differ markedly [10,11] and, in many instances, only synthetic, codon-usage adapted, genes provide significant yields. The availability of a core suite of distinct efficient host-broad host range expression cloning vector systems, differing in their expression specificities, should enable determination of optimal production systems for different proteins.

*Pseudomonas putida *strain KT2440 is a metabolically versatile soil bacterium with considerable potential in a broad range of diverse industrial and environmental applications [12]. Its certification as a biosafety strain [13,14], its ability to express a broad spectrum of foreign proteins at high levels and the availability of powerful customized tools for genetic analysis and manipulation [15], make KT2440 an important prokaryotic cell factory. These features suggest that it might be a useful production system for antibody fragments.

In this study, we have assessed the potential of KT2440 for the soluble production of different recombinant scFvs namely, the model murine anti-hen egg-white lysozyme scFv, D1.3 [11,16-19], and two phage display-selected human scFvs: TOB5-D4 [[11], Al-Halabi *et al. *in preparation], directed against C-reactive protein (CRP) [20], an inflammation indicator in human blood, and HT186-D11 [21], directed against mucin1 (MUC1), a diagnostic marker and potential therapeutic target of cancer [22,23].

## Results and Discussion

### Expression plasmids and synthetic genes

The key features of the antibody expression plasmids constructed in this study are shown in Table 1. They were generated using new synthetic RK2 broad host range plasmid-based chassis developed by the group of Victor de Lorenzo (in preparation) carrying either the inducible *Ptac *[13,24] or TOL plasmid *xyl *operon *Pm *[25,26] promoters. We further modified the chassis by equipping it with the G10L ribosome binding site (RBS) with epsilon enhancer [27] and the *Erwinia carotovora pelB *leader sequence [28] to effect the export of expressed polypeptides to the periplasm. The scFv format consists of variable regions of the antibody heavy and light chains (VH and VL, respectively), joined by a 15-25 amino acid linker [29]. Polynucleotide sequences encoding Myc-HIS_6_-Strep-Tag^® ^II or HIS_6_-Myc-Strep-Tag^® ^II affinity tags were placed downstream of the VH-linker-VL coding sequences (Table 1) to facilitate purification and detection of the scFvs in western blots and enzyme-linked immunosorbent assays (ELISA). In addition, synthetic scFv genes were generated to assess the influence of codon usage adaptation on expression levels in *P. putida *(Table 1). The original scFv gene constructs were expressed from the *Ptac *promoter, whereas the synthetic gene constructs were expressed from either *Ptac *or *Pm *promoters (Table 1). Although synthetic codon usage adapted gene constructs of TOB5-D4 (anti-CRP) and HT186-D11 (anti-mucin1) scFvs were readily generated, for unknown reasons the equivalent version of D1.3 (anti-lysozyme) scFv could not be synthezised by the commercial supplier. For generation of the native sequence constructs, the RBS and Strep-tag^® ^II were added using primer overhangs.

**Table 1:** Properties of the RK2-based broad-host range plasmid constructs with the *Ptac/lacIq *(pSEVAlac) or *Pm/xylS *(pSEVAxyl) promoter/regulator gene systems used in this study.

Construct	scFv specificity	Promoter	Affinity-tags	codon optimization^1^	% GC^2^
pSEVAlacD1.3n	hen egg-white lysozyme	*Ptac*	HIS6/Myc/Strep-tag^® ^II	_	52,3
pSEVAlacTOB5-D4n	human CRP	*Ptac*	Myc/HIS6/Strep-tag^® ^II	_	55,0
pSEVAlacTOB5-D4s	human CRP	*Ptac*	Myc/HIS6/Strep-tag^® ^II	Jcat/Eurofins MWG Operon	68,3
pSEVAxylTOB5-D4s	human CRP	*Pm*	Myc/HIS6/Strep-tag^® ^II	Jcat/Eurofins MWG Operon	68,3
pSEVAlacHT186-D11n	human mucin1	*Ptac*	HIS6/Myc/Strep-tag^® ^II	_	57,8
pSEVAlacHT186-D11s	human mucin1	*Ptac*	HIS6/Myc/Strep-tag^® ^II	GeneOptimizer^®^/GENEART	67,6
pSEVAxylHT186-D11s	human mucin1	*Pm*	HIS6/Myc/Strep-tag^® ^II	GeneOptimizer^®^/GENEART	67,6

^1^Synthetic scFv determinants with optimized codon usage: - indicates the original scFv sequence, Jcat/Eurofins MWG Operon and GeneOptimizer^®^/GENEART indicates a codon adaptation tool and a supplier; ^2 ^%GC content of the scFv coding sequence.

### Affinity isolation of scFvs

**Figure 1:**
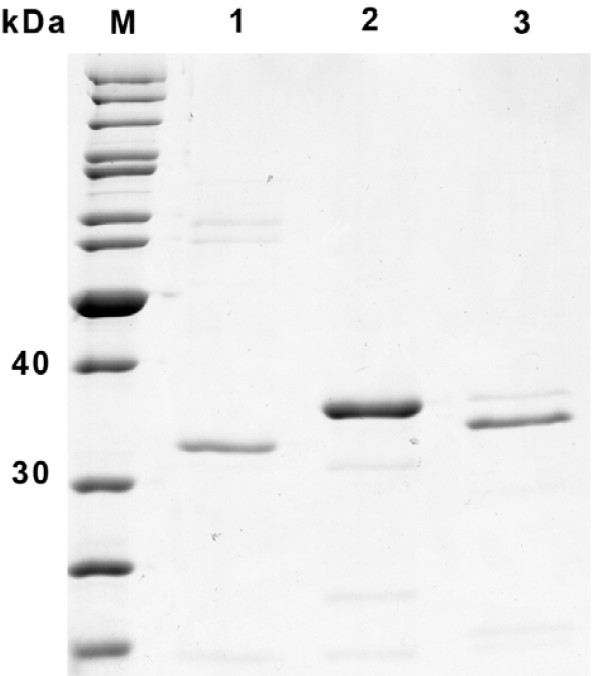
**Purity of single-step affinity-purified scFvs from KT2440**. Proteins were isolated, affinity purified, incubated and subjected to freeze:thaw cycles to assess their stability in elution buffer, as described above and run on a 12.5% SDS polyacrylamide gel. M = PAGE Ruler unstained protein molecular weight marker (Fermentas); 1, D1.3; 2, TOB5-D4 (anti-CRP), 3, HT186-D11 (anti-MUC1).

Different combinations of cell lysis and affinity purification strategies were tested in order to determine a rapid, simple isolation/purification protocol. Purification of hexahistidine-tagged scFvs from clarified whole cell extracts by immobilized metal ion affinity chromatography (IMAC) resulted in a protein mixture containing three major species between 30-50 kDa, of which the mid band was confirmed by N-terminal sequencing to be the scFv. The addition of a second affinity chromatography on Strep-Tactin Superflow in series (tandem) resulted in scFv fragments of high purity with no visible background in sensitively Coomassie Blue-stained SDS-PAGE (data not shown). Single step purification on Strep-Tactin Superflow resin in gravity flow columns, however, proved sufficient for most purposes, including yield determination. This single-step protocol yielded scFv fragments with a purity of ~95%. Incubation of the purified fragments for 16-24 h on ice, followed by at least one freeze thaw cycle in elution buffer, did not cause significant degradation of the scFvs (Figure 1). Lysis of cells by sonication or use of the bacterial protein extraction reagent (B-Per, Thermo fisher scientific), or both in combination, gave similar results, which is perhaps not surprising, given that the scFvs are targeted to the periplasmic space. Moreover, purification of selectively released periplasmic proteins (see below) by single step Strep-Tactin Superflow chromatography resulted in a purity comparable to those obtained by the tandem strategy.

### scFv yield optimization

#### Induction

**Figure 2:**
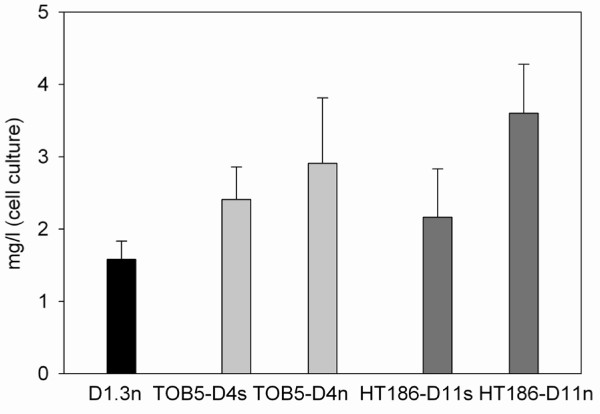
**Yields of soluble scFvs from KT2440 constructs**. Mean values and standard deviations are given of yields from single-step purified soluble scFvs obtained from 3-6 independent expression and purification experiments.

**Figure 3:**
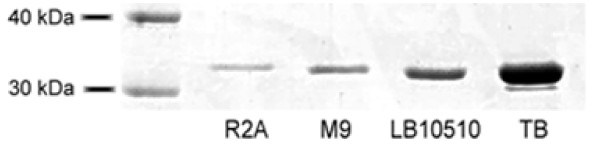
**Effect of different culture media on HT186-D11 production**. SDS-PAGE of single step purified scFvs from periplasmic extractions of *P. putida *KT2440 cultures expressing scFvs from construct HT186-D11n on different media.

Two transcriptional expression systems based on the *Ptac *and *Pm *promoters, that are widely used to express heterologous genes, were assessed for their efficacy in scFv production by KT2440 in otherwise identical RK2 plasmid-based expression constructs (Table 1). In the case of the TOB5-D4s and HT186-D11s scFvs, yields from the *Ptac *promoter constructs were more than twice those from the *Pm *promoter constructs. Optimal yield conditions determined for *Ptac *constructs were 3 h induction by 1 mM IPTG at 30°C in LB-medium, which gave yields of 1.5 mg/l for the D1.3 scFv, 2.9 mg/l for the anti-CRP scFv, and 3.6 mg/l for the HT186-D11 scFv (Figure 2; yield means of 3-6 individual expression experiments involving 100 ml cultures agitated at 180 rpm, and isolation by single step affinity purification on 1 ml Strep-Tactin Superflow gravity columns). The influence of medium composition on yield was also assessed and it was found that yields decreased in the order: Terrific Broth (TB) > LB > M9 (15 mM succinate) > R2A (Figure 3), indicating that enriched, buffered media favour scFv production and that medium optimization has considerable potential for yield enhancement.

#### Codon usage optimization

KT2440 is an expression host with a high average GC content (~61.5%) and a codon usage preference distinct from those of the scFv determinants used in this study [14]. To assess to which extent adaptation of the scFv codons to those preferred by KT2440 could influence scFv yields, synthetic scFv determinants composed of the preferred codons were designed *in silico*, synthesized and tested in the expression systems. Although synthetic versions of the TOB5-D4 (anti-CRP) and HT186-D11 (anti-MUC1) scFv sequences were made, it was not possible to obtain a synthetic version of the D1.3 (anti-lysozyme) scFv (Table 1). For the two former scFvs, it was found that the yields - 2.4 mg/l for TOB5-D4s (anti-CRP) and 2.1 mg/l for HT186-D11s (anti-MUC1) (Figure 2) - were lower than those from the original constructs, so in these cases, codon usage optimization did not increase expression levels. This might reflect the ability of KT2440 to efficiently express a broad range of foreign genes, or limitation of higher levels of expression otherwise attainable due to the higher than normal (for KT2440) GC contents of the synthetic genes (Table 1), or sub-optimal mRNA secondary structures created by the changes [30].

The key question arising from these results is how the scFv yields obtained compare with those obtainable from existing expression systems. Unfortunately, pertinent information could not be found in the literature, and values that are available are not usable due to differences in antibody and/or experimental procedures followed. To obtain relevant information on this issue, we introduced the broad range constructs into the *Escherichia coli *expression strain BL21 (DE3). Yields of the TOB5-D4n and HT186-D11n scFvs from *E. coli *were 2.5 to 4-fold lower than those from KT2440, and, as might be predicted, even lower in the case of the high GC synthetic genes (data not shown). Literature yields in *E. coli *for an anti-CRP scFv antibody of 0.55 mg/l [31] and the D1.3 scFv antibody of 0.29 mg/l [10] lie within the same low range.

### Periplasmic export

**Figure 4:**
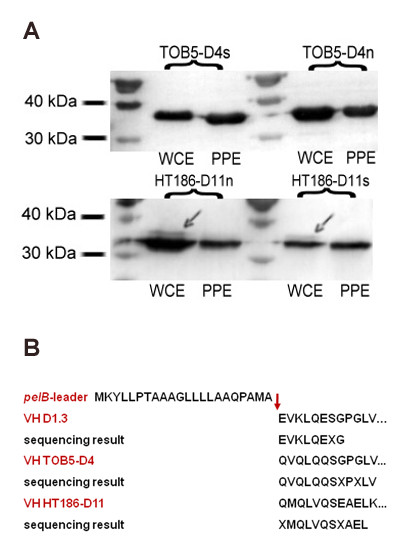
**Periplasmic export of scFvs and signal peptide cleavage**. A.) Western-blot of purified antibodies extracted from whole cells (WCE) and periplasmic preparations (PPP) and chromogenic detection of the Strep-tag^® ^II at the C-terminus. A minor fraction of incomplete N-terminal processing (cleavage of the 22 amino acid *pelB*-leader sequence) is visible only in the case of HT186-D11 whole cell extracts (arrows). B.) N-terminal sequencing by Edman degradation confirmed the predicted cleavage sites following the A-M-A motif. X, amino acid position with ambiguous identification.

All genetic constructs were designed to create precise amino-terminal translational fusions of the scFv-coding sequences with the *E. carotovora pelB *signal sequence to target the scFvs for secretion by the Sec pathway. For assessment of the efficiency of processing of the scFv fragments produced in KT2440, and their translocation to the periplasm, periplasmic fractions were isolated. This procedure generally gave scFv yields in the same range as, though with greater variability than, those obtained by whole cell lysis. To ascertain whether the increased variation in yield is due to incomplete release of periplasmic proteins during the isolation procedure or incomplete translocation to the periplasm, perhaps resulting from overloading of the secretion apparatus, Sec-mediated proteolytic cleavage of the *pelB *leader sequences of the scFvs was analyzed by electrophoretic separation followed by western blot detection of the C-terminal Strep-tag^® ^II (Figure 4A). As expected, only fully processed scFvs were detected in periplasmic preparations, indicating that periplasmic scFvs are completely processed. In the case of proteins extracted from whole cells, only fully processed mature anti-CRPs were detectable, although small amounts of unprocessed HT186-D11n and HT186-D11s scFvs were identified (Figure 4A, compare Figure 1), indicating that at the moment of sampling small amounts of untransported protein were still in the cytoplasm. The ratio of processed:unprocessed polypeptide did not seem to correlate with expression levels, since unprocessed protein was also observed in cells carrying the lower expressing HT186-D11s construct. More likely is the possibility that the VH-coding amino acid sequence affects the secondary structure around the cleavage junction and negatively impacts on the efficiency of processing. The variable yields in periplasmic extracts probably reflect incomplete disruption of the outer membrane by the procedure we used, but might also result from partial non-specific leakage of periplasmic proteins to the medium, a phenomenon previously observed for antibody fragments exported by means of the PelB leader peptide in *E. coli *[32]. It should, nevertheless, be emphasized that this procedure for selective isolation of periplasmic proteins has the major advantage of yielding active, soluble antibody fragments uncontaminated by cytoplasmic proteins.

The signal peptide cleavage sites of the recombinant scFvs were predicted by means of the *in silico *tool Predisi [33] to be located between amino acids 22 and 23, directly after the A-M-A motif, which is also the preferred sequence motif (A-X-A) for interaction with signal peptidase I [34], and experimentally confirmed by N-terminal sequencing of isolated antibody (Figure 4B). Other sites of cleavage were not detected, which indicates that the KT2440 LepB peptidase (PP_1432), which shares 39% amino acid identity and 52% similarity with the *E. coli *K-12 peptidase, precisely recognizes and cleaves the PelB processing site.

### Antibody activity

**Figure 5:**
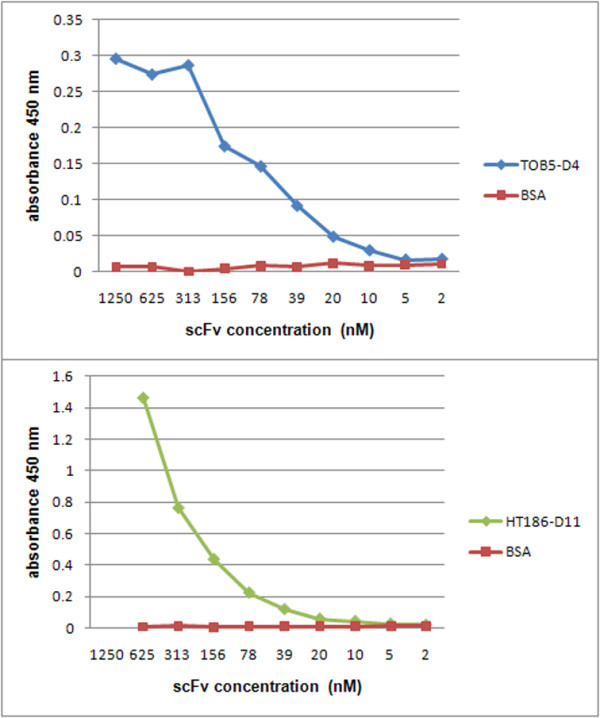
**Specific binding activities of the scFvs produced in KT2440**. The binding activities of the scFvs to cognate antigens was measured by ELISA. The scFv concentration detection limits were ~10 nM. Absorbance was measured at 450 nm and corrected for absorbance at 620 nm.

In order to assess whether the extracted periplasmic scFv polypeptides were correctly folded and had acquired correct binding activity, the binding of the TOB5-D4 and HT186-D11 antibodies to their cognate antigens were measured by ELISA assays using plates coated with 100 ng per well of either CRP antigen (BiosPacific, Emeryville, USA), MUC1 32 aa peptide with a C-terminal cysteine (APDTRPAPGSTAPPAHGVTSAPDTRPAPGSTA-C)[21], or bovine serum albumin (BSA; controls). A primary mice anti-Myc-tag IgG, combined with a secondary Fab-specific goat anti-mice IgG horseradish peroxidase (HRP) conjugate were used for detection of bound antibody, as previously described [35]. As can be seen in Figure 5, high specific antigen binding was observed for both recombinant scFvs, with significant signals recorded till antibody concentrations of 10 nM, which corresponds to dilutions of 1:256, for an antibody having an initial concentration of 2.5 μM. Recombinant anti-CRP antibody preparations obtained from both whole cells and periplasmic extracts had similar binding activities (data not shown), which is consistent with the findings shown in Figure 4A and the correct folding of periplasmic antibody.

## Conclusions

We have presented here an assessment of the potential of *Pseudomonas putida *KT2440 as a cell factory for the production of soluble recombinant antibody fragments that bind to antigens of high interest for diagnostics and therapy. With the described construct design, good yields of soluble, active scFvs are obtained through simple extraction of periplasmic polypeptides and single step affinity purification. These yields considerably exceeded those obtained from equivalent constructs in *E. coli *K-12, so KT2440 would seem to be a promising cell factory for recombinant antibody fragment production. It is likely that high density fed-batch cultivation of KT2440 [36], which are typically characterized by cell densities of up to more than 100 g/l, will allow achievement of much higher yields by this host. A common means of optimizing expression levels of recombinant proteins is the use of synthetic genes with host-optimized codon usage. In our study, this approach did not achieve increased expression, possibly due to the expression versatility of this host for foreign genes. However, the possibility that a less rigorous adaptation that maintains the *P. putida *KT2440 natural % G+C value might give better expression would seem worth exploring in future, especially in conjunction with other optimization efforts involving exploration of media and cultivation conditions as well as genetic engineering and genome streamlining approaches. The broad host range feature of the constructs presented here will of course allow their testing in other host systems and potential discovery of even more powerful cell factories for antibody production.

## Methods

### Vector construction

Expression construct pSEVAlacTOB5-D4n was generated by amplification of the scFv sequence from antibody expression vector pOE101-TOB5-D4 [[11], Al-Halabi *et al. *in preparation], using primers A (fw 5'-GCGAATTCTTAACTTTAAGAAGGAGATATATCC ATG A AATACCTATTGCCTACGGC-3') and B (rev 5'-GCTCTAGA TTACTTTTTCGAACTGCGGGTGGCTCCA ATGATGATGGTGATGATGGGATAGATCTTC-3') and cloning via *Eco*RI and *Xba*I to pSEVA-RK2-Sm-lac (pSEVA424). Vector pSEVAlacHT186-D11n was generated amplifying HT186-D11 from phage display vector pHAL14-HT186-D11 [11,21] using primers A and C (5'-GCTCTAGA TTACTTTTCGAACTGCGGGTGCGACCATGCGGCCCCATTCAGATCC TCT TCTGAGATG-3'), and cloning via EcoRI and XbaI into pSEVA-RK2-Sm-lac. Plasmid pSEVAlacD1.3n was constructed using the same primers and restriction sites for the amplification product of pHAL14-D1.3 [11]. Sequences for ribosome binding sites [27], restriction sites and the Strep-tag^® ^II [37] were added with the corresponding primer overlaps.

Vector pSEVAlacTOB5-D4s and pSEVAxylTOB5-D4s were generated using an *in silico*-designed synthetic construct incorporating the RBS and Strep-tag^® ^II. The sequence was codon-usage adapted to that of *P. putida *KT2440, using Jcat [38], and synthesized by Eurofins MWG (Ebersberg, Germany). The construct was cloned into pSEVA-RK2-Sm-lac and pSEVA-RK2-Sm-XylPm via *MfeI*/*Eco*RI and *XbaI*. A synthetic construct was also generated from HT186-D11, codon usage adapted to *P. putida*, and synthesized by GENEART (Regensburg, Germany). Cloning in pSEVA-RK2-Sm-lac and pSEVA-RK2-Sm-XylPm *via *EcoRI/HindIII resulted in pSEVAlacHT186-D11s and pSEVAxylHT186-D11s respectively. All constructs contained the 66 bp *Erwinia carotovora pelB*-leader sequence for periplasmic export and are summarized in Table 1. *E. coli *strain DH5α, chemicals and enzymes for PCR and cloning were purchased from Fermentas (St. Leon-Rot, Germany) and New England Biolabs (Ipswich, MA, USA). Integrity of the constructs was verified by sequencing the synthetic construct prior to cloning, and restriction digestion after cloning. Native sequences were amplified from sequence validated templates [11,21], using high fidelity proof-reading polymerase (Phusion™) and confirmed by restriction digestion after cloning.

### Transformation of *Pseudomonas putida *KT2440

Competent cells were prepared using buffer containing 300 mM sucrose as described before [39]. 30 ng of plasmid constructs were used to transform 40 μl of competent *P. putida *KT2440 (DSM 6125) cells by electroporation, which was carried out in prechilled 2 mm cuvettes using a Gene Pulser II with pulse controller plus and capacitance extender plus (Bio-Rad, Hempel Hempstead, UK). Cell:DNA mixes were pulsed at 2.5 kV, 25 μF and 200-500Ω resistance, and subsequently plated on selection medium containing 100 μg/ml of streptomycin and spectinomycin.

### Production of scFvs with *Pseudomonas putida *KT2440

An 50 ml overnight liquid culture of the *P. putida *KT2440 clone freshly transformed with the respective construct (Table 1) was used to inoculate the production culture 1:100. Production test-cultures were grown in 200 ml cultures at 150 rpm at RT, 30°C and 37°C. Luria Bertani (LB) (5 g/l yeast extract, 10 g/l tryptone, 10 g/l NaCl), rich Luria Bertani Broth (10 g/l yeast extract, 10 g/l tryptone, 10 g/l NaCl), M9 containing 15 mM succinate, TB, and R2A [40,41] containing 50 μg/ml streptomycin were used in 500 ml baffled Erlenmeyer flasks. In the mid logarithmic phase (at OD_595 _of ~0.5-0.6), production was induced by addition of isopropyl-ß-D-thiogalactopyranoside (IPTG) at concentrations ranging between 100 μM and 1 mM, or 15 mM 3-methylbenzoate, for pSEVAxyl-TOB5-D4s and pSEVAxyl-HT186-D11s. Final yields reported in Figure 2 are from lysis of cells cultured in 100 ml LB at 180 rpm and 30°C. The cells were harvested by centrifugation.

### Production of scFvs in *E. coli*

To compare yields of recombinant antibody fragments from *P. putida *KT2440 with those from *E. coli *K-12, we introduced the pSEVAlacTOB5-D4 and pSEVAlacHT186-D11 constructs containing the *tac *promoter for expression into *E. coli *strain BL21 (λDE3) (Stratagene). K-12 and KT2440 constructs were cultured at RT for HT186-D11n and 30°C for TOB5-D4n with other parameters being as described for KT2440, which in initial experiments also gave the best antibody yields in *E. coli.*

### Periplasmic export and signal peptide cleavage

To obtain periplasmic extracts, harvested cells were resuspended in PE buffer (20% (w/v) sucrose, 50 mM Tris, 1 mM EDTA, pH 8), incubated for 30 min on ice with brief vortexing every 5 minutes, and centrifuged at 20,000 rcf for 30 min.

Completeness of signal peptide cleavage was assessed by electrophoresis of antibody fragments on 12.5% SDS-PAGE, semi-dry transfer to PVDF membranes (Peqlab), and anti-Strep-tag detection using Strep-Tactin alkaline phosphatase (AP) conjugate (IBA, Göttingen, Germany) and chromogenic BCIP (5-Bromo-4-Chloro-3'-Indolyphosphate p-Toluidine Salt), NBT (Nitro-Blue Tetrazolium Chloride) detection (AP Blue Membrane Substrate Solution, Sigma). The cleavage position was determined by N-terminal sequencing by Edman degradation and compared to the *in silico *predicted sites obtained with the Predisi tool [33].

### Affinity purification of antibody fragments

The proteins were obtained by whole cell lysis by sonication in sonication buffer (50 mM Tris, 100 mM NaCl, 5 mM MgCl_2_, 0,05% (v/v) Triton X-100, pH 8,0), bacterial protein extraction reagent B-Per (Thermo Fischer scientific) according to the manufacturer's instructions, sonication in B-Per or by periplasmic extraction (see below). Whole cell extracts were centrifuged for 15 min at 15,000 rcf to obtain cell-free extracts which, like periplasmic preparations were applied directly to affinity chromatography columns.

We used IMAC on Ni-TED (Machery-Nagel), for HIS-tag-based affinity purification, or Strep-Tactin Superflow (IBA) columns for Strep-tag^® ^II-based affinity purification, or both in sequence. The washing and elution procedures were carried out according to the manufacturers' recommendations. Protein concentrations of purified scFv solutions were determined by the method by Gill and van Hippel [42], using the individually calculated molar extinction coefficients for the processed amino acid sequences without signal peptides (Protein Calculator v3.3, http://www.scripps.edu/~cdputnam/protcalc.html), and the UV absorbance at 280 nm (protein) and 260 nm (as control for possible nucleic acid contamination), determined with the NanoDrop^® ^ND-1000 spectrophotometer (Peqlab) or lab spectrometer (Eppendorf).

### Antigen binding ELISA

The antigens, in PBS, were used to coat wells of 96-well microtitre plates (Maxisorp, Nunc). After coating overnight at 4°C, the wells were washed three times with PBST, and blocked with 2% (w/v) skim milk powder in PBST (2% M-PBST) for 1.5 h at room temperature, followed by three washes with PBST. For the ELISA, soluble scFvs were diluted in 100 μl 2% M-PBST and incubated in the antigen-coated plates for 1.5 h at room temperature, followed by three PBST washes. Bound scFvs were detected with the murine mAb (9E10, Sigma), which recognises the C-terminal c-myc tag, and a goat anti-mouse serum, conjugated with horseradish peroxidase (HRP) (Sigma; 1:10,000). For visualisation the chromogenic substrate 3,3',5,5'-tetramethylbenzidine (TMB) was added. The staining reaction was stopped by addition of 100 μl 1 N sulphuric acid and absorbance at 450 nm and 620 nm was measured using a SUNRISE™ microtitre plate reader (Tecan, Crailsheim, Germany).

## Abbreviations used

aa: amino acid; AP: alkaline phosphatase; RBS: ribosome binding site; CRP: human C-reactive protein; EDTA: ethylenediaminetetraacetic acid; ELISA: enzyme-linked immunosorbent assay; Fab: fragment antigen binding; HRP: horseradish peroxidase; IgG: immunglobulin G; IMAC: immobilized metal ion affinity chromatography; IPTG: isopropyl-β-D-thiogalactopyranoside; MUC1: human mucin1; PBS(T): phosphate-buffered saline (tween); PVDF: polyvinylidene fluoride, RPM: rotations per minute; rcf, relative centrifugal force; scFv: single chain fragment variable; VH: antibody variable domain of the heavy chain; VL: antibody variable domain of the light chain.

## Competing interests

MH and SD are inventors on a patent application regarding anti-MUC1 antibodies (PCT/EP2009/005218). This does not alter our adherence to all the Microbial Cell Factories policies on sharing data and materials. The other authors declare that they have no competing interests.

## Authors' contributions

TD designed and coordinated the study, drafted the manuscript, performed the experiments with lab support of SCK and analyzed the data. MS conducted the antigen ELISA, MH and SD provided the scFv sequences, MH, SD and KNT helped to draft the manuscript and to design the study. All authors read and approved the final manuscript.
